# 免疫检查点抑制剂在*EGFR*突变型晚期非小细胞肺癌中的应用

**DOI:** 10.3779/j.issn.1009-3419.2022.102.32

**Published:** 2022-09-20

**Authors:** 玉军 郑, 巍 姜, 晶 李, 璐璐 代, 东妍 陈, 颜君 李, 磊 黄, 明吉 王

**Affiliations:** 116001 大连，大连市友谊医院 The Friendship Hospital of Dalian, Dalian 116001, China

**Keywords:** 分子特征, 免疫检查点抑制剂, 肺肿瘤, Molecular characteristics, Inmune checkpoint inhibitors, Lung neoplasms

## Abstract

近年来，免疫检查点抑制剂（immune checkpoint inhibitors, ICIs）极大地提高了无驱动基因突变的非小细胞肺癌（non-small cell lung cancer, NSCLC）患者的生存率。与野生型肿瘤相比，表皮生长因子受体（epidermal growth factor receptor, *EGFR*）突变的肿瘤在程序性细胞死亡配体1（programmed cell death ligand 1, PD-L1）、肿瘤突变负荷（tumor mutational burden, TMB）等免疫微环境特征上具有更大的异质性。ICIs是否适用于*EGFR*突变的NSCLC患者一直存在争议。临床研究显示免疫单药对于*EGFR*突变的NSCLC患者无显著疗效，ICIs联合化疗和抗血管生成药物则显示了良好的生存获益。本文就*EGFR*突变晚期NSCLC患者ICIs单药或联合治疗的临床研究及相关机制进行综述。

## Abstract (en)

In recent years, immune checkpoint inhibitors (ICIs) have greatly improved the survival rate of non-small cell lung cancer (NSCLC) patients without driver mutation. Compared with wild-type tumors, tumors with epidermal growth factor receptor (*EGFR*) mutations have greater heterogeneity in immune microenvironment characteristics such as programmed cell death ligand 1 (PD-L1) and tumor mutational burden (TMB). Whether ICIs is suitable for NSCLC patients with *EGFR* mutation has been controversial. Clinical studies have shown that immunomonotherapy has no significant effect on patients with *EGFR* mutant NSCLC. ICIs combined with chemotherapy and antiangiogenic drugs show good survival benefits. This paper overviews the clinical research and related mechanism of ICIs single drug or combination therapy inadvanced NSCLC patients with *EGFR* mutation.

流行病学研究^[1]^发现，2022年中国新发癌症病例482万，癌症死亡321万；肺癌仍是最常见的癌症，也是主要的癌症死亡原因。近十年来，晚期NSCLC的精准治疗，包括靶向治疗、免疫治疗取得了极大的进展，明显延长了患者的无进展生存期（median progression-free survival, mPFS）和总生存期（median overall survival, mOS），并且提高了生活质量。精准治疗的前提是精准诊断，分子分型是非小细胞肺癌（non-small cell lung cancer, NSCLC）实施靶向治疗的前提。随着第二代测序技术（next generation sequencing, NGS）的广泛应用，越来越多的驱动基因被发现，目前针对表皮生长因子受体（epidermal growth factor receptor, EGFR）、间变性淋巴瘤激酶（anaplastic lymphoma kinase, ALK）、ROS1、MET、RET、KRAS、HER2和BRAF等靶点的药物已获得相应适应证；但靶向治疗获得较高疗效的同时不可避免地会出现耐药和肿瘤复发^[2]^。同时，免疫检查点抑制剂（immune checkpoint inhibitors, ICIs）彻底改变了Ⅲ期和Ⅳ期NSCLC的治疗格局。KEYNOTE-024研究^[3]^确立了单药ICIs在程序性细胞死亡配体1（programmed cell death ligand 1, PD-L1）表达≥50%患者中的一线治疗地位；PACIFIC研究^[4]^开启了Ⅲ期NSCLC放化疗后免疫巩固治疗时代；KEYNOTE-042研究^[5]^进一步将ICIs单药一线治疗标准扩大至PD-L1表达≥1%；KEYNOTE-189^[6]^和KEYNOTE-407^[7]^研究表明无论PD-L1表达水平，ICIs联合化疗均可带来获益。

免疫治疗是否可以为驱动基因阳性NSCLC患者带来获益？目前尚不能将所有驱动基因变异的NSCLC归为一类，不同驱动基因的驱动性、相应靶向药物的疗效、肿瘤免疫微环境（tumor immune microenvironment, TIME）等均有差异；一方面，IMMUNOTARGET研究^[8]^发现不同驱动基因阳性的晚期NSCLC患者对免疫治疗的响应并不相同，优先靶向还是优先免疫仍存在争议；另一方面，也有研究^[9-12]^指出，免疫和靶向治疗序贯的顺序、间隔时间与严重不良反应的发生率有关。*EGFR*突变是NSCLC的一个独特亚型，在全球及我国是最常见的类型，对EGFR酪氨酸激酶抑制剂（EGFR-tyrosine kinase inhibitors, EGFR-TKIs）具有显著敏感性。因此研究也最广泛，本文就免疫治疗在*EGFR*突变阳性晚期NSCLC患者中的应用进行综述。

## 1 *EGFR*突变晚期NSCLC免疫治疗临床研究

### 1.1 *EGFR*突变晚期NSCLC免疫单药治疗

Lisberg等^[13]^的一项Ⅱ期临床研究应用帕博利珠单抗（Pembrolizumab，200 mg/3 wk，共35个周期）一线未经TKIs治疗的晚期*EGFR*阳性、PD-L1阳性（≥1%，包括8例PD-L1≥50%）NSCLC患者，主要终点为客观缓解率（objective response rate, ORR）。原计划入组25例患者，在入组了11例后，因缺乏疗效而停止了入组。在这11例患者中，只有1例因标本错误判为*EGFR*阳性而实际为野生型的患者疗效为部分缓解（partial response, PR），其余10例均未观察到疗效。表明免疫单药不适合*EGFR*突变晚期NSCLC的一线治疗。因此，在多数的一线ICIs的Ⅲ期临床研究中均排除了*EGFR*突变型NSCLC患者，如CheckMate 026^[14]^（纳武利尤单抗 *vs* 化疗）、KEYNOTE-024^[2]^（帕博利珠单抗 *vs* 化疗）、KEYNOTE-042^[5]^（帕博利珠单抗 *vs* 化疗）、Impower 110^[15]^（阿特珠单抗 *vs* 化疗）。

ICIs在后线治疗中的机会如何？2016年美国癌症研究协会（American Association of Cancer Research, AACR）报告了美国麻省总院晚期NSCLC 22例*EGFR*突变和6例*ALK*融合型，中位治疗线数为3（0-8），结果显示：*EGFR*/*ALK*阳性患者接受ICIs的ORR为3.6%，明显低于*EGFR*/*ALK*阴性患者的23.3%^[16]^。Chee等^[17]^荟萃分析汇总了CheckMate 017^[18]^、CheckMate 057^[19]^、KEYNOTE-010^[20]^、OAK^[21]^和POPLAR^[22]^等5项研究，评价ICIs和多西他赛单药二线治疗晚期非鳞NSCLC患者的疗效，结果显示在总体人群（*n*=1, 903）和EGFR野生型人群（*n*=1, 362）中，ICIs能显著延长OS（*P* < 0.000, 1），但在*EGFR*突变型（*n*=186）中，并没有改善OS，差异也无统计学意义（HR=1.05, 95%CI: 0.70-1.55, *P* < 0.81）。一项Ⅱ期研究（WJOG8515L）^[23]^观察纳武利尤单抗（Nivolumab）（*n*=52）或卡铂-培美曲塞治疗*EGFR*突变获得性耐药非T790M突变晚期NSCLC的疗效，结果提示Nivolumab组的中位PFS（median PFS, mPFS）和1年PFS概率分别为1.7个月和9.6%，对照组分别为5.6个月和14.0%[*P* < 001；危险比（hazard ratio, HR）为1.92]。OS分别为20.7个月和19.9个月（HR=0.88, 95%CI: 0.53-1.47），ORR分别为9.6%和36.0%。亚组分析显示，具有高TMB、T细胞炎症基因表达谱得分高、细胞毒性T细胞浸润或其募集相关的基因表达高，Nivolumab获益明显。基于此得出结论，在总体人群中，与卡铂-培美曲塞相比，Nivolumab不会延长PFS。基因表达谱可以鉴定出与Nivolumab疗效相关的TIME。因此，对于EGFR-TKIs治疗失败的NSCLC患者二线治疗，与化疗相比，ICIs单药也并未显示出明显改善生存的优势。ATLANTIC^[24]^是使用度伐利尤单抗（Durvalumab）单药治疗*EGFR*/*ALK*阳性NSCLC患者三线以上的单药前瞻性研究，结果提示PD-L1≥25%的患者mOS为13.3个月；PD-L1≥25%且*EGFR*阳性患者mOS为16.1个月；12个月OS率为53.3%；24个月OS率为40.7%。

### 1.2 *EGFR*突变晚期NSCLC免疫联合治疗

免疫治疗联合靶向治疗的一项I期临床研究^[25]^探索了Nivolumab联合厄洛替尼（Erlotinib）治疗晚期NSCLC患者的效果；共入组21例晚期NSCLC患者，20例EGFR-TKIs耐药，1例初治；研究结果显示，mPFS为5.1个月，mOS为18.7个月；而且整体耐受性可。该结果为一代EGFR-TKIs耐药后患者的治疗提供了新的思路，但由于入组病例数偏少，目前尚无法推广并应用于临床实践。TATTON^[9]^研究探索奥希替尼（Ositinib）联合Selumetinib、Savolitinib或Durvalumab治疗*EGFR*突变晚期NSCLC患者的最佳剂量和安全性。研究中期发现，联合治疗组间质性肺炎的发生率明显升高，因此，认为奥希替尼联合Durvalumab的方案不可行。

ICIs联合化疗是一种潜在的有益策略，2019世界肺癌大会（World Conference on Lung Cancer, WCLC）上发表的一项^[26]^特瑞普利单抗联合化疗用于EGFR-TKIs治疗失败的*EGFR*突变阳性T790M阴性晚期NSCLC患者的Ⅱ期临床研究结果：ORR为54.8%，疾病控制率（disease control rate, DCR）为93.7%，整体人群PFS达7.6个月；3级以上不良事件发生率为51.4%。因此，*EGFR*突变阳性晚期NSCLC免疫治疗联合化疗尚需要大规模临床研究。

关于免疫联合免疫的治疗模式，KEYNOTE-021^[27]^试验队列D（剂量发现队列）和H（剂量扩展队列）探讨了在晚期NSCLC的后线治疗中帕博利珠单抗加伊普利单抗（Ipilimumab）的益处，与CheckMate 227^[28]^一线Nivolumab加Ipilimumab的观察结果相反，免疫联合疗效有限，10例*EGFR*突变患者中只有1例记录了PR。

尽管PD-1/PD-L1单抗在上述试验中多数以失败告终，那是不是就以为免疫治疗在*EGFR*突变的患者人群中没有一席之地呢？IMpower150^[29]^研究验证了以下假设：ICIs联合化疗和抗血管生成药物治疗晚期NSCLC（包括*EGFR*突变患者）更有效。在对TKIs耐药后的*EGFR*突变亚组中，与贝伐珠单抗加化疗组相比，在贝伐珠单抗加化疗组中再联合阿替利珠单抗（Atelizumab）可改善ORR，延长PFS和OS（ORR：71% *vs* 42%；mPFS：10.2个月 *vs* 6.9个月，HR=0.61；mOS：未达到 *vs* 18.7个月，HR=0.61）。有了这些令人鼓舞的结果，ICIs加上抗血管生成药物再加化疗的新组合对于*EGFR*突变的晚期NSCLC的二线治疗是一种有希望的策略。

总之，ICIs单药作为*EGFR*突变晚期NSCLC的后线治疗效果有限。联合治疗包括ICIs联合化疗，尤其是Atelizumab+贝伐珠单抗+卡铂+紫杉醇的四重疗法，疗效显著；但由于不良事件发生率高，在应用时需要特别小心。

另外，需要注意ICIs与TKIs序贯治疗的顺序，近来不断有研究指出，ICIs序贯TKIs可能导致严重不良反应。一项回顾性研究^[10]^分析了ICIs序贯Ositinib的安全性，结果显示，15%（6/41）的患者发生了严重的免疫相关不良反应（immune related adverse reactions, irAE）。并且，严重irAE的发生率与ICIs和TKIs的间隔时间有关，分别为24%（5/21）（距最后一剂ICIs < 3个月）、13%（1/8）（3个月-12个月）和0%（0/12）（> 12个月）。然而，在Ositinib序贯ICIs或ICIs序贯其他EGFR-TKIs时并未发现严重irAE。

## 2 *EGFR*阳性晚期NSCLC免疫特征

目前，*EGFR*突变NSCLC患者ICIs效果不佳的机制认为，相比EGFR野生型，*EGFR*突变的NSCLC肿瘤特点是免疫惰性表型，具有低PD-L1表达、低肿瘤突变负荷（tumor mutational burden, TMB）和低肿瘤浸润淋巴细胞（tumor infiltrating lymphocytes, TILs）。此外，单细胞分析显示，EGFR-TKIs无论是原发性还是继发性耐药肿瘤，CD73表达均上调。EGFR信号通路和EGFR-TKIs在许多方面都影响免疫疗效。

### 2.1 *EGFR*突变型NSCLC中PD-L1表达

一项包含3, 283例患者的荟萃分析^[30]^显示，*EGFR*突变型NSCLC比野生型PD-L1表达更低，为了证实这项荟萃分析的结果，研究者分析了癌症基因组图谱（The Cancer Genome Atlas, TCGA）和国内广东肺癌研究所（Guangdong Lung Cancer Institute, GLCI）数据库中PD-L1的蛋白质和mRNA图谱。相比于EGFR野生型，mRNA图谱显示*EGFR*突变NSCLC中PD-L1及TILs均低表达；免疫组化显示T细胞浸润程度亦低。联合分析发现*EGFR*突变组的双阳性（PD-L1^+^/CD8^+^ TIL）比例明显减少，而双阴性（PD-L1^-^/CD8^-^ TIL）比例增加。同时，用85例肺腺癌的全基因组测序进行样本验证，发现*EGFR*突变阳性NSCLC患者的TMB较野生型低。

PD-L1作为一种免疫检查点蛋白，在肿瘤细胞和TILs中均有表达^[31]^。PD-L1的表达受两种不同机制的影响：内在表达和获得性表达。内在表达方式是通过*EGFR*突变激活下游信号通路从而上调肿瘤细胞中PD-L1的表达，如丝裂原活化蛋白激酶/细胞外信号调节激酶/c-Jun（MAPK/ERK/c-Jun）、Hippo/Yes相关蛋白（Hippo/YAP）和Janus激酶/信号转导子和转录激活子3（JAK/STAT3）信号通路^[32-34]^。相反，体外研究^[35]^表明，EGFR通路激活可以抑制γ干扰素（interferon-γ, IFN-γ）活性从而抑制获得性PD-L1表达。

研究结果的差异性可能与多种因素有关，如不同的PD-L1检测技术（不同的抗体、检测平台和不同的阳性阈值）、肿瘤异质性和肿瘤组织来源（如细胞学标本、存档标本、新鲜标本、原发和转移部位）。

### 2.2 *EGFR*突变型NSCLC的TMB

TMB被定义为整个肿瘤基因组中体细胞突变总数，是预测ICIs疗效的新兴生物标志物。与EGFR耐药/未知组相比，*EGFR*敏感性突变（根据对第一代EGFR-TKIs的反应定义）的TMB显著降低。Haratani等^[36]^评估Nivolumab对于*EGFR*突变的NSCLC患者的疗效，TMB中位数为101，对Nivolumab有显著反应的患者的TMB显著高于无反应的患者。此外，Dong等^[30]^发现，与EGFR野生型组相比，*EGFR*突变组（外显子19 Del、L858R、L861Q、G719X和S768I）的TMB中位数显著降低（56 *vs* 181）。TMB降低可能是*EGFR*突变患者对ICIs反应不佳的机制^[30, 37, 38]^。然而，TMB的检测、计算方法和阈值尚无统一标准。进一步确定TMB作为ICIs生物标志物可能有助于选择合适的人群。

### 2.3 *EGFR*突变型NSCLC的TIME

TIME是肿瘤生长发育的内部环境，有研究已经报道，*EGFR*突变可以调节TIME状态，从而影响抗肿瘤免疫反应，包括TILs、Tregs、髓源性抑制细胞（myeloid-derived suppressor cells, MDSCs）、肿瘤相关巨噬细胞（tumor associated macrophage, TAM）、免疫调节细胞因子和外泌体。

TILs是一组存在于肿瘤巢和间质中的肿瘤浸润性和抗原细胞群。在TIME的发生发展过程中充当抗肿瘤免疫细胞，通过释放IFN-γ、穿孔素和颗粒酶B等细胞因子破坏肿瘤细胞；TILs的数量决定了抗肿瘤的杀伤效率。CXC趋化因子配体10（CXC chemokine ligand 10, CXCL10）可以通过磷脂酰肌醇3-激酶（phosphatidylinositol 3-kinase, PI3K）/蛋白激酶B（protein kinase B, AKT）信号通路招募效应CD8^+^ T细胞^[39, 40]^。EGFR通路激活下调CXCL10，从而抑制效应CD8^+^ T细胞的募集^[41]^。因此，*EGFR*突变的肿瘤通常表现为CD8^+^ TILs浸润较低^[31, 42]^，导致免疫缺陷和不良预后^[36]^。Toki等^[43]^利用荧光技术探索了*EGFR*突变与TILs状态之间的关系中发现，*EGFR*突变组的患者中检测到衰竭或休眠免疫的状态（高CD3、低Ki67和低颗粒酶B），PD-L1高表达的肿瘤细胞和基质细胞具有高度浸润的活化TILs（分别为*P*=0.001, 4和*P*=0.02）。此外发现，不同*EGFR*突变位点免疫学特征存在差异：*EGFR* L858R样本中CD8^+^ T细胞的表达显著高于*EGFR*外显子19缺失，但肿瘤细胞和基质细胞中PD-L1表达两者无差异^[43, 44]^。

Tregs被认为是抗肿瘤免疫的一个关键障碍，Treg在*EGFR*突变的肿瘤中高度浸润^[45]^，通过分泌白细胞介素-10（interleukin-10, IL-10）、IL-35和转化生长因子β（transforming growth factor β, TGF-β），减弱自然杀伤（natural killer, NK）细胞、CD4^+^ T细胞和CD8^+^ T细胞介导的抗肿瘤免疫反应^[46]^。临床前研究^[47]^观察到*EGFR*突变可以通过JNK-C-Jun途径上调C-C类趋化因子（C-C class chemokines 22, CCL22），从而上调Treg相关基因的表达，并招募Treg细胞。Wang等^[48]^发现，EGFR/糖原合成酶激酶3（glycogen synthase kinase 3, GSK-3）/叉头盒蛋白3（forkhead box protein p3, Foxp3）轴通过双调蛋白（amphiregulin, AREG）介导Treg的免疫抑制功能，促进肿瘤进展。双调蛋白是EGFR配体之一，能够促进肿瘤进展^[49]^。此外，Huang等^[50]^发现含有EGFR的外泌体促进树突状细胞（dendritic cell, DC）分泌吲哚胺2,3-双加氧酶（indoleamine 2,3-dioxygenase 1, IDO），进而促进CD3^+^CD4^+^CD25^+^ T细胞向Treg的转化。

其他影响TIME的因素还有主要组织相容性复合体（major histocompatibility complex, MHC），其在抗原提呈中起重要作用。有研究^[51-53]^表明，MHC-Ⅰ和MHC-Ⅱ的表达通过IFN-γ信号通路和下游MEK/ERK信号通路下调。另外，*EGFR*突变的肿瘤细胞可能上调CD73^[44]^。CD73是多种肿瘤和免疫细胞表达的负性免疫调节因子，它可将三磷酸腺苷（adenosine triphosphate, ATP）转化为腺苷（adenosine, ADO），ADO与免疫细胞上的ADO受体结合，上调Treg的表达，介导肿瘤细胞的转移和增殖。丰富的ADO对多种免疫细胞具有免疫抑制活性。它促进Treg的激活和髓来源的抑制性细胞（myeloid-derived suppressor cells, MDSCs）的积累，进一步减弱效应T细胞、自然杀伤（natural killer, NK）细胞和DC的抗肿瘤功能，使巨噬细胞（macrophages, Mφ）极化向M2倾斜，并抑制Teff介导的抗肿瘤反应，介导肿瘤免疫逃逸。体外实验^[53]^中，EGF诱导CD73表达，EGFR-TKIs抑制CD73表达。这些发现表明，异常的EGFR信号增加了CD73的表达，并且CD73-ADO轴是*EGFR*突变肿瘤中的另一种可能的免疫抑制机制。Le等^[54]^证明，*EGFR*突变型肺癌的小鼠模型中，阻断CD73-ADO轴可显著抑制肿瘤进展。

### 2.4 EGFR-TKIs对肿瘤免疫微环境的影响

抗EGFR治疗可改变TME，理论上及体外细胞系实验表明，EGFR-TKIs通过抑制EGFR信号下调PD-L1的表达^[55]^。然而，一些临床分析^[56, 57]^发现，经EGFR-TKIs治疗后，PD-L1表达呈上升趋势且PD-L1高表达的患者比低表达的患者有更长的OS（7.1个月 *vs* 1.7个月，*P*=0.003, 3）。Justin等^[16]^也证明，EGFR-TKIs耐药后，21%的患者肿瘤组织中PD-L1表达增加。一项血液分析^[58]^也发现EGFR-TKIs治疗1周后，血液中的PD-L1^+^ T细胞显著增加。EGFR-TKIs原发性耐药的患者表现出肿瘤细胞PD-L1表达和PD-L1^+^CD8^+^ T细胞浸润^[59, 60]^。研究^[34, 46, 61]^发现EGFR-TKIs可以减轻EGFR信号对T细胞的抑制，削弱Treg细胞的功能，增强IFN-γ的产生，并增强MHC-Ⅰ和MHC-Ⅱ的表达。小鼠模型中，Brea等^[62]^证明了EGFR-TKIs的动态效应，他们观察到EGFR-TKIs对TIME的影响在早期是有益的，但在后期是免疫抑制的。在早期，CD8^+^ T细胞、DC和M1样肿瘤相关巨噬细胞（tumor associate macrophage, TAM）的数量呈增加趋势，而Treg浸润减少。EGFR-TKIs治疗的后期，IL-10和CCL2分泌增加促进了骨髓间充质干细胞的迁移和激活，从而抑制免疫，促进血管生成和转移^[47, 61]^。短期低剂量Erlotinib导致*EGFR*突变肿瘤的免疫介导细胞毒性以及NK细胞和抗原特异性T细胞的肿瘤溶解。然而，在长期使用Erlotinib后，这种增强的免疫介导的细胞毒性消失了^[61]^。上述研究为在Erlotinib耐药之前给予ICIs和Erlotinib联合治疗提供了理论依据。

## 3 *EGFR*基因突变位点与ICIs的有效性

一项研究^[63]^发现，在600例*EGFR*突变的NSCLC患者中，49例（8.2%）罕见突变（G719X、L861Q、S768I和Ex20-ins），PD-L1^+^者占49.0%（24/49），敏感突变（19del和L858R）患者为12.2%（67/551）（*P* < 0.05）。PD-L1^+^与相对较短的OS相关（15.2个月 *vs* 29.3个月，*P*=0.006），此外，PD-L1^+^主要见于CD8^+^ TILs浸润的肿瘤（*P*=0.001）。EGFR罕见突变同时PD-L1^+^和CD8^+^ T细胞浸润的患者占36.7%，双阳性组的预后最差（*P*=0.023）。值得注意的是，PD-L1^+^和CD8^+^ T细胞双阳性的患者对ICIs疗效好。因此得出结论：罕见*EGFR*突变的NSCLC患者伴随PD-L1^+^和CD8^+^ TILs的比率高。另一项研究^[39]^发现，EGFR T790M阴性和T790M阳性患者的PFS分别为2.1个月和1.3个月（*P*=0.099, HR=0.48, 95%CI: 0.20-1.24）。PD-L1表达水平≥1%和 < 1%患者的mPFS分别为2.1个月和1.3个月（*P*=0.084, HR=0.37, 95%CI: 0.10-1.21）。PFS随着PD-L1表达水平的增加而增加，域值分别为≥10%和≥50%。在T790M阴性患者中，PD-L1高表达的比例高于T790M阳性患者。免疫治疗应答者的CD8^+^ TILs密度和非同义突变负荷显著较高。在EGFR-TKIs治疗后，*EGFR*突变阳性且T790M阴性NSCLC患者更可能受益于免疫治疗，这可能是因为PD-L1表达水平高于T790M阳性患者。

## 4 小结

免疫治疗并不适合作为*EGFR*突变的NSCLC患者的一线治疗。PD-L1的低表达、TMB的低水平以及免疫抑制环境的上调是*EGFR*突变NSCLC患者ICIs治疗处于劣势的原因。不同*EGFR*突变亚型（外显子19 *vs* 外显子21 *vs* 少见突变 *vs* T790M）TIME的特征不同。对于EGFR-TKIs治疗失败的患者，与化疗相比，免疫单药治疗并未显示出明显改善生存的优势。EGFR-TKIs对TIME的影响是动态的。EGFR-TKIs与ICIs联合应用不仅没有提高疗效，反而增加了毒性。然而，免疫治疗和化疗的结合显示了初步的疗效，抗血管治疗也能改变免疫微环境，IMpower150^[29]^中ICIs联合化疗和抗血管生成药物显示了良好的生存益处。及时动态监测，选择合适的时间窗，可以扩大适合免疫治疗的人群。总体而言，联合治疗可能更适合*EGFR*突变阳性TKIs耐药后晚期NSCLC的患者。驱动基因阳性肺癌免疫治疗的未来是优化人群、选择时机和方案。
